# The relationships between West Nile and Kunjin viruses.

**DOI:** 10.3201/eid0704.010418

**Published:** 2001

**Authors:** J H Scherret, M Poidinger, J S Mackenzie, A K Broom, V Deubel, W I Lipkin, T Briese, E A Gould, R A Hall

**Affiliations:** Department of Microbiology and Parasitology, University of Queensland, Brisbane, Queensland 4072, Australia.

## Abstract

Until recently, West Nile (WN) and Kunjin (KUN) viruses were classified as distinct types in the Flavivirus genus. However, genetic and antigenic studies on isolates of these two viruses indicate that the relationship between them is more complex. To better define this relationship, we performed sequence analyses on 32 isolates of KUN virus and 28 isolates of WN virus from different geographic areas, including a WN isolate from the recent outbreak in New York. Sequence comparisons showed that the KUN virus isolates from Australia were tightly grouped but that the WN virus isolates exhibited substantial divergence and could be differentiated into four distinct groups. KUN virus isolates from Australia were antigenically homologous and distinct from the WN isolates and a Malaysian KUN virus. Our results suggest that KUN and WN viruses comprise a group of closely related viruses that can be differentiated into subgroups on the basis of genetic and antigenic analyses.


### The Relationships between West Nile and Kunjin Viruses

**Jacqueline H. Scherret,* Michael Poidinger,† John S. Mackenzie,* Annette K. Broom,‡ Vincent Deubel,§ W. Ian Lipkin,¶ Thomas Briese,¶ Ernest A. Gould,# and Roy A. Hall***

*University of Queensland, Brisbane, Queensland, Australia; †Bioinformatics Inc., Eveleigh, New South Wales, Australia; ‡ University of Western Australia, Nedlands, Western Australia, Australia; §Institut Pasteur, Paris, France; ¶University of California, Irvine, California, USA; and #Institute of Virology and Environmental Microbiology, Oxford, England

Until recently, West Nile (WN) and Kunjin (KUN) viruses were classified as distinct types in the *Flavivirus* genus. However, genetic and antigenic studies on isolates of these two viruses indicate that the relationship between them is more complex. To better define this relationship, we performed sequence analyses on 32 isolates of KUN virus and 28 isolates of WN virus from different geographic areas, including a WN isolate from the recent outbreak in New York. Sequence comparisons showed that the KUN virus isolates from Australia were tightly grouped but that the WN virus isolates exhibited substantial divergence and could be differentiated into four distinct groups. KUN virus isolates from Australia were antigenically homologous and distinct from the WN isolates and a Malaysian KUN virus. Our results suggest that KUN and WN viruses comprise a group of closely related viruses that can be differentiated into subgroups on the basis of genetic and antigenic analyses.

Kunjin (KUN) and West Nile (WN) viruses belong to the Japanese encephalitis (JE) antigenic complex of the *Flavivirus* genus in the family Flaviviridae (1). The *Flavivirus* genus comprises >70 antigenically related, positive-stranded RNA viruses (2,3). KUN and WN viruses are maintained in a natural transmission cycle involving mosquito vectors and bird reservoir hosts, with humans and horses believed to be incidental hosts (4,5). Clinical symptoms most commonly associated with infection with KUN and WN viruses include febrile illness or mild encephalitis. WN virus has been associated with fatal cases of acute meningoencephalitis and fulminant hepatitis (6).

Early cross-neutralization studies with polyclonal antisera raised to single strains of WN and KUN viruses revealed that these viruses shared a close relationship but were antigenically distinct (7-9). This close relationship was also shown genetically by Coia et al. (10), who compared the sequence of the MRM61C KUN isolate with that of a Ugandan strain of WN (WNFCG) (11-13) and showed that the nucleotide and amino acid sequence identity between the two viruses was 82% and 93%, respectively, in the coding region of the genome. Although genetic studies have shown that KUN virus exists in Australia as a single topotype with <2% nucleotide divergence (14,15), Berthet et al. (16) demonstrated that WN viruses were divided into two lineages. Although these comparisons demonstrated a close relationship between the two viruses, further sequence information is needed from additional isolates of both viruses to fully establish their phylogenetic association within the genus. This report describes the results of sequence analyses of 31 Australian KUN isolates; a KUN isolate from Sarawak, Malaysia; and 28 WN isolates from Africa, India, Europe, and New York (Tables 1 and 2). These virus isolates had all been identified as WN or KUN virus by traditional antigenic means. The Koutango (KOU) isolate was also included in this study, as it belongs to the JE serogroup and is closely related to the KUN/WN group of viruses (9,17).

Address for correspondence: Roy A. Hall, Department of Microbiology and Parasitology, University of Queensland, Brisbane, Queensland 4072, Australia; fax: 61-7-33654620; e-mail: royboy@biosci.uq.edu.au

### Materials and Methods

### Virus and Cell Culture

Virus strains sequenced in this study are listed with their sources of isolation in Table 1. African green monkey (Vero) cells were grown at 37°C in M199 (Gibco, New York) with 20 mM HEPES (Gibco) and supplemented with 2% L-glutamine and either 10% fetal bovine serum (FBS) for growth or 2% FBS for maintenance. Viruses were cultured in Vero cells by inoculating cell monolayers with virus at a multiplicity of infection of 1. Infected cell culture supernatants were harvested when ≥70% of the cells exhibited cytopathic effect. Infected supernatant was clarified by centrifugation at 2000 x *g* at 4°C for 15 min, and aliquots were stored at -70°C. A line of *Aedes albopictus* (C6/36) cells was cultured in M199 without HEPES and supplemented with FBS for growth or maintenance, as described. The cells were incubated at 28°C in a humidified atmosphere with 5% $CO_2$.

### Reverse Transcription-Polymerase Chain Reaction (RT-PCR) and Sequencing

A single-step RT-PCR procedure (22) was performed on each virus isolate. The region amplified within the envelope

Table 1. West Nile and Kunjin virus isolates and nucleotide sequences used in this study

| Virus ID | Year of isolation | Source of isolation | Place of isolation | GenBank Accession Number |
|---|---|---|---|---|
| KUN35911 | 1984 | Horse brain | Hunter Valley, NSW,[a] AU | AF196511 (E gene) |
| KUNP1553[b] | 1994 | *Culex* sp. | Marble Bar, WA, AU | AF297856 (NS5/3'UTR)<br>AF196495 (E gene) |
| KUNCH16465C | 1974 | *Cx. ann.* | CH, Qld, AU | AF297841 (NS5/3'UTR)<br>AF196504 (E gene) |
| KUNCH16514C | 1974 | *Cx. ann.* | CH | AF297842 (NS5/3'UTR)<br>AF196501 (E gene) |
| KUNCH16532C | 1974 | *Cx. ann.* | CH | AF297843 (NS5/3'UTR)<br>AF196513 (E gene) |
| KUNCH16549E | 1974 | *Cx. ann.* | CH | AF297844 (NS5/3'UTR)<br>AF196520 (E gene) |
| KUNM695 | 1982 | *Cx. ann.* | Victoria, AU | AF297852 (NS5/3'UTR)<br>AF196496 (E gene) |
| KUNM1465 | 1983 | *Cx. ann.* | Victoria, AU | AF297851 (NS5/3'UTR)<br>AF196522 (E gene) |
| KUNMRM5373 | 1966 | *Oriolus flavocintus* (bird) | MRM, Qld, AU | AF297859 (NS5/3'UTR)<br>AF196509 (E gene) |
| KUNMRM16 | 1960 | *Cx. ann.* | MRM | AF196505 (E gene) |
| KUNMRM61C | 1960 | *Cx. ann.* | MRM | AF196516 (E gene) |
| KUNOR130 | 1973 | *Cx. ann.* | OR, East Kimberley, WA, AU | AF297857 (NS5/3'UTR)<br>AF196492 (E gene) |
| KUNOR134 | 1973 | *Cx. ann.* | OR | AF196506 (E gene) |
| KUNOR166 | 1973 | *Cx. ann.* | OR | AF196499 (E gene) |
| KUNOR205 | 1973 | *Aedes tremulus* | OR | AF297858 (NS5/3'UTR)<br>AF196515 (E gene) |
| KUNOR354 | 1974 | *Cx. ann.* | OR | AF297855 (NS5/3'UTR)<br>AF196518 (E gene) |
| KUNOR393 | 1974 | *Cx. ann.* | OR | AF196503 (E gene) |
| KUNOR4 | 1972 | *Cx. ann.* | OR | AF196523 (E gene) |
| KUNCX255 | 1982 | *Cx. ann.* | Wyndham, East Kimberley | AF297845 (NS5/3'UTR)<br>AF196514 (E gene) |
| KUNCX238 | 1982 | *Cx. ann.* | Wyndham, East Kimberley | AF196502 (E gene) |
| KUNBoort | 1984 | Horse spinal cord | Boort, Victoria, AU | AF297840 (NS5/3'UTR)<br>AF196519 (E gene) |
| KUNFC15 | 1986 | *Cx. ann.* | West Kimberley, WA, AU | AF297846 (NS5/3'UTR)<br>AF196510 (E gene) |
| KUNHu6774 | 1991 | Human | Southern NSW, AU | AF297847 (NS5/3'UTR)<br>AF196493 (E gene) |
| KUNK6547 | 1991 | *Cx. ann.* | SE Kimberley, WA, AU | AF196521 (E gene) |
| KUNK1738 | 1989 | *Cx. ann.* | OR | AF297848 (NS5/3'UTR)<br>AF196494 (E gene) |
| KUNK5374 | 1989 | *Cx. ann.* | SE Kimberley, WA, AU | AF297849 (NS5/3'UTR)<br>AF196517 (E gene) |
| KUNK2499 | 1984 | *Cx. ann.* | OR | AF196498 (E gene) |
| KUNK6590 | 1991 | *Cx. ann.* | Broome, West Kimberley, WA, AU | AF297850 (NS5/3'UTR)<br>AF196500 (E gene) |
| KUNSH183 | 1991 | Chicken | Victoria, AU | AF297853 (NS5/3'UTR)<br>AF196491 (E gene) |
| KUNWK436 | 1979 | *Cx. ann.* | Camballin, West Kimberley, WA, AU | AF297854 (NS5/3'UTR)<br>AF196507 (E gene) |
| KUNV407 | 1983 | *Cx. ann.* | Jabiru, NT, AU | AF196508 (E gene) |
| KUNMP502-66 | 1966 | *Cx. pseudovishnui* | Sarawak, Borneo, Malaysia | AF196534 (E gene) |
| HB6343 | 1989 | Human | CAR | AF196542 (NS5/3'UTR)<br>AF196528 (E gene) |
| ArTB3573 | 1982 | Tick | CAR | AF196541 (NS5/3'UTR)<br>AF196527 (E gene) |
| MgAn798 | 1978 | *Coracopsis vasa* (bird) | Madagascar | AF196543 (NS5/3'UTR) |
| 63134Ent 280 | <1963 | Human | Uganda | AF196539 ( NS5/3'UTR)<br>AF196530 (E gene) |
| ArA1Dj | 1968 | Mosquito | Algeria | AF196536 (NS5/3'UTR)<br>AF196529 (E gene) |
| ArNa1047 | unknown | Mosquito | Kenya | AF196535 (NS5/3'UTR) |
| G2266 | 1955 | *Cx. vishnui* | Sathuperi, India | AF196537 (NS5/3'UTR)<br>AF196525 (E gene) |
| G22886 | 1958 | *Cx. vishnui* | Sathuperi, India | AF196538 (NS5/3'UTR)<br>AF196524 (E gene) |
| 804994 | 1980 | Human brain biopsy | Bangalore Field Station, Karnataka, India | AF196540 (NS5/3'UTR)<br>AF196526 (E gene) |
| Sarafend | unknown | unknown | unknown | AF196533 (E gene) |
| KOU DakAad 5443 | 1968 | *Tatera kempi* (rodent) | Senegal, Africa | AF196532 (E gene) |

[a]NSW = New South Wales; AU = Australia; WA = Western Australia; *Cx. ann.* = *Culex annulirostris;* CH = Charleville; Qld = Queensland; MRM = Mitchell River Mission; OR = Ord River; NT = Northern Territory; CAR = Central African Republic; UTR = untranslated region.
[b]P1553 was isolated from a culture of C6/36 cells inoculated with culture fluid derived from a mosquito pool from which Edge Hill (EH) virus had also been isolated (Annette Broom, pers. comm.).

Table 2. Additional West Nile and Kunjin virus sequences included in this study

| Virus ID | Year of isolation | Source of isolation | Place of isolation | GenBank Accession Number | Region of genome | Reference |
|---|---|---|---|---|---|---|
| KUNMP502-66 | 1966 | *Culex pseudovishnui* | Sarawak | L49311 | NS5/3'UTR | 17 |
| NY99 | 1999 | *Phoenicopterus chilensis* (Chilean flamingo) | NYC[a] | AF196835 | E | 18 |
| NY99 | 1999 | Human | NYC | AF202541 | NS5/3'UTR | 21 |
| ISR98 | 1998 | Goose | Israel | AF205882 | E | V. Deubel,unpub. data |
| Rom96 | 1996 | Human | Romania | AF130363 | E | 19 |
| Rom97-50 | 1997 | Unknown | Romania | AF130362 | E | 20 |
| ArB310 | 1967 | *Culex* sp. | CAR | AF001566 | E | 16 |
| Mor96 | 1996 | Unknown | Morocco | AF205884 | E | V. Deubel,unpub. data |
| Italy98 | 1998 | Unknown | Italy | AF205883 | E | V. Deubel,unpub. data |
| ArD93548 | 1993 | *Cx. neavei* | Senegal | AF001570 | E | 16 |
| AnD27875 | 1979 | *Galago senegalensis* | Senegal | AF001569 | E | 16 |
| PaH651 | 1965 | Human | France | AF001560 | E | 16 |
| AnMg798 | 1978 | *Coracopsis vasa* (bird) | Madagascar | AF001559 | E | 16 |
| ArMg978 | 1988 | *Cx. univittatus* | Madagascar | AF001574 | E | 16 |
| MP22 | unknown | unknown | Uganda | AF001562 | E | 16 |
| UGA-B956 | unknown | unknown | Uganda | AF208017 | NS5 | 21 |
| ArD78016 | 1990 | *Aedes vexans* | Senegal | AF001556 | E | 16 |
| HB83P55 | 1983 | Human | CAR | AF001557 | E | 16 |
| Eg101 | 1951 | Human | Egypt | AF001568 | E | 16 |
| Eg101 | 1951 | Human | Egypt | AF260968 | NS5 | Bowen et al., unpub. data |
| ArA3212 | 1981 | *Cx. guiarti* | Ivory Coast | AF001561 | E | 16 |
| KUNMRM16 | 1960 | *Cx. ann.* | MRM | L48979 | NS5/3'UTR | 17 |
| KUNMRM61C | 1960 | *Cx. ann.* | MRM | L48978 | NS5/3'UTR | 17 |
| Sarafend | unknown | unknown | unknown | L48977 | NS5/3'UTR | 17 |
| KOUDakAad 5443 | 1968 | *Tatera kempi* (rodent) | Senegal | L48980 | NS5/3'UTR | 17 |
| WNFCG | 1937 | Human | Uganda | M12294 | E and NS5/3'UTR | 11 |

[a]NYC = New York City; *Cx. ann.* = *Culex annulirostris;* CAR = Central African Republic; MRM = Mitchell River Mission; UTR = untranslated region.

(E) gene used the primers KUN5276 (GCG TGT GGT TCT TCA AAC TCC A) and WN4752 (TGC GTG TCC AAC CAT GGG TGA AGC) with the isolates Sarafend, MP502-66, and a strain of KOU virus, DakAad 5443. Primer KUN5276 was used with primer KUN4778 (ATA ATG ACA AGC GGG CTG ACC C) for the remaining isolates. The region of the virus genome encompassing the terminus of the nonstructural protein, NS5 and the 5' end of the 3' untranslated region (3'UTR), was amplified by using the previously published universal flavivirus PCR primers EMF1 and VD8 (23).

Both strands of the PCR product were then sequenced on a 377 automated sequencer (Applied Biosystems International [ABI], Foster City, CA, USA) by using the same primer pair. The two sequences derived from each PCR product were initially aligned by using the program SeqEd (ABI) and a consensus sequence determined. The consensus sequences were then aligned by using the program Clustal W (24), and results were further analyzed by using phylogenetic programs in Bionavigator (http://www.bionavigator.com). Percentage nucleotide similarity was calculated by the Old Distance (GCG) program, and bootstrap confidence levels were calculated with 1,000 replicates by using the Consense program (25). Sequences determined in this study have been deposited in GenBank (National Institutes of Health, Bethesda, MD, USA) (Table 1). Additional sequences included in this analysis are listed in Table 2.

### Enzyme-Linked Immunosorbent Assay (ELISA)

Antigenic profiles of each isolate were compared by using a panel of anti-KUN monoclonal antibodies (MAbs) (26,27) and anti-WN MAbs (28,29) in ELISA as described (26). All MAbs were produced to the E protein except for 3.1112G, which was specific for the NS1 protein.

### Results

### Genetic Analysis

In accordance with previous reports (16,18,21), the phylogenetic trees generated from both E gene and NS5/3'UTR sequences grouped most of the isolates into two major lineages (Figures 1 and 2). Australian KUN isolates and WN isolates from North, West, and central Africa; southern and eastern Europe; India; the Middle East; and New York constituted lineage I. Lineage II comprised WN isolates from West, central, and East Africa and Madagascar. Genetic lineage was not significantly associated with date or source of isolation, with most isolates of both lineages coming from human, mosquito, and avian sources between 1950 and 1990. However, as noted, all viruses isolated during outbreaks of human or avian disease in the last decade belonged to lineage I. Lineage I viruses grouped together with an average sequence identity of 80% (E gene) and 77% (NS5/3'UTR), while the viruses of lineage II contained a single cluster with an average identity of 82% and 83%, respectively. The lineage I viruses were further separated into three clusters: the Australian KUN isolates; the Indian WN viruses; and WN isolates from Africa, the Middle East, Europe, and North America. The divergence observed between lineage I and lineage II viruses was in the range of 16.5% to 30.8% and 19% to 36.5% for sequences of the E gene and NS5/3'UTR, respectively. High bootstrap confidence levels (100%) for the sequences of the NS5/3'UTR also support the separation of the

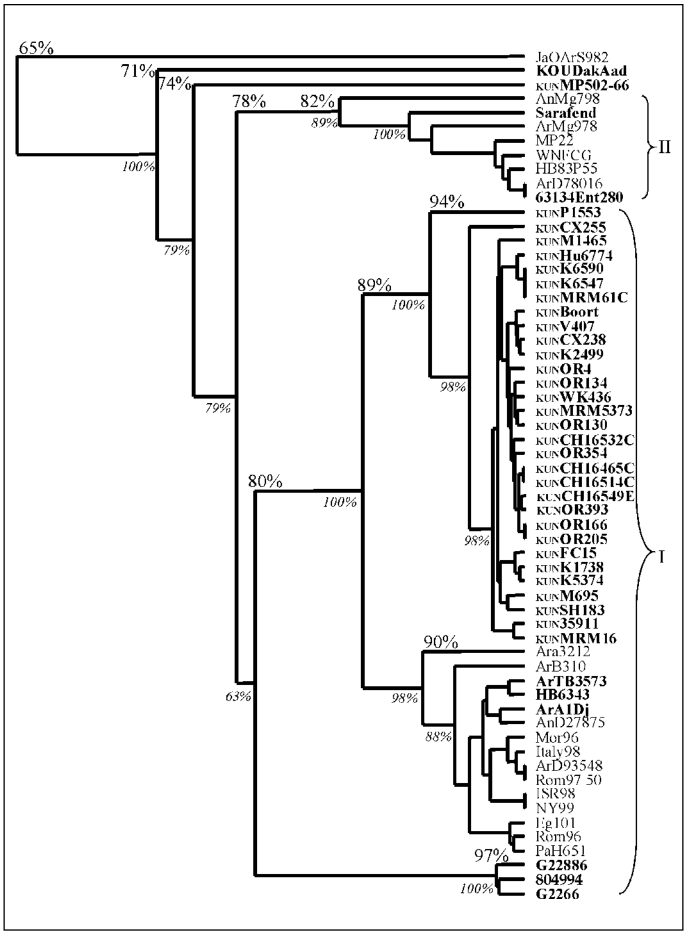

Figure 1. Phylogenetic tree constructed by the neighbor-joining algorithm based on E gene nucleic acid sequence data. Numbers above branches indicate average percentage nucleotide similarity between limbs, while the values in italics indicate the percentage bootstrap confidence levels. Isolates highlighted in bold are sequences obtained in this study. Dendrogram outgrouped with the Japanese encephalitis isolate, JaOArS982 (30; GenBank Accession Number M18370).

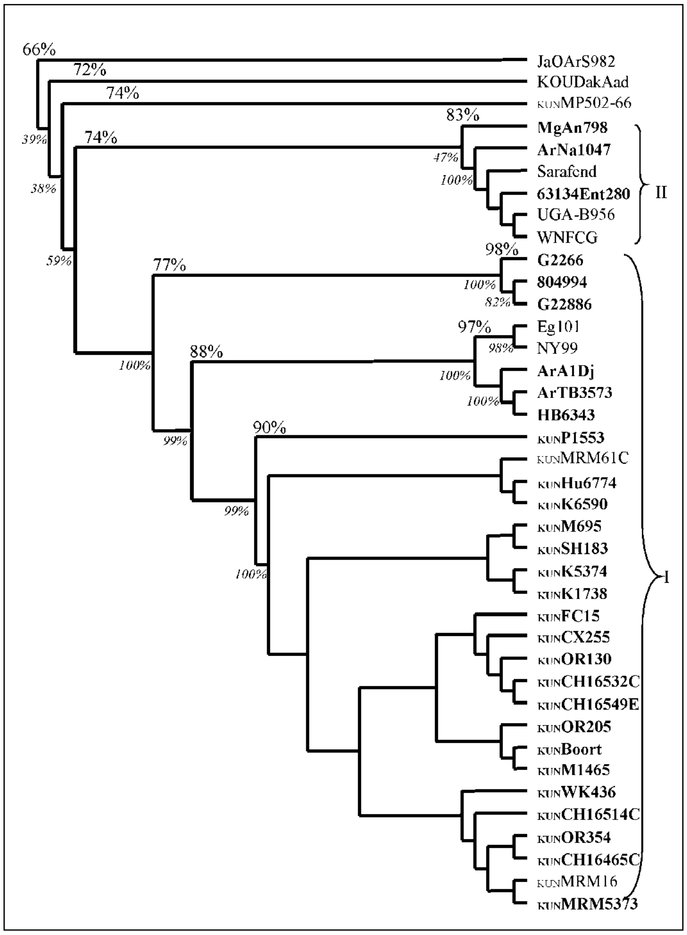

Figure 2. Phylogenetic tree constructed using the neighbor-joining algorithm based on nucleic acid sequence data encompassing the 3' end of the NS5 gene and 5' end of the 3' UTR untranslated region). Numbers above branches indicate average percentage nucleotide similarity between limbs, while the values in italics indicate the percentage bootstrap confidence levels. Isolates highlighted in bold are sequences obtained in this study. Dendrogram outgrouped with the JE isolate, JaOArS982 (30; GenBank Accession Number M18370).

two lineages and the branching of the NY99 cluster of WN viruses with the Australian KUN viruses in lineage I, rather than with the WN group of viruses in lineage II. The clustering of the Indian WN group in lineage I based on sequences in the E gene, however, was at a lower bootstrap confidence level (63%).

The sequence of the virus from Malaysia, KUN MP502-66, grouped outside the two lineages described. Similarly, the KOU virus, which was 72%-73% identical to KUN MP502-66, did not group with either lineage. The range of percentage divergence between KUN MP502-66 and KOU viruses with the lineage I and lineage II viruses (Table 3) shows that these two isolates display similar divergence from all other isolates in this study, supporting their grouping outside the two main lineages.

The viruses of lineage I group together in three tight clusters. The first of these includes the Australian KUN viruses, which were 94% identical when sequences of the E gene were compared and 90% when the sequences of the NS5/3'UTR were compared. High bootstrap confidence levels (100% for sequences from the E gene and 99% for sequences from the NS5/3'UTR) separated the Australian KUN viruses from the other isolates. However, extremely low bootstrap confidence levels were observed for most of the branches between the Australian KUN viruses in both dendrograms, which also suggests that these viruses are closely related and cannot be definitively separated from each other. The Indian viruses also cluster together, with a sequence identity of 97% and 98% for sequences of the E gene and NS5/3'UTR, respectively. The WN isolates in the remaining cluster of lineage I are 90% and 97% identical, respectively, for the regions sequenced. When compared with the Australian KUN isolates, this cluster, which includes the 1999 New York isolate, shared a sequence identity of 89% for the E gene and

Table 3. Range of percentage divergence between the Malaysian and Koutango isolates with lineage I and lineage II viruses

| | E gene | | NS5/3'UTR | |
|---|---|---|---|---|
| | Lineage I | Lineage II | Lineage I | Lineage II |
| MP502-66 | 20%-30% | 20%-30% | 21%-35% | 21%-25% |
| KOU | 25%-30% | 29%-32% | 26%-39% | 22%-25% |

UTR = untranslated region.

88% for the NS5/3'UTR. Similarly, when the sequences of the Australian KUN isolates were compared with those of the WN Indian viruses, they were 80% identical for the E gene and 77% identical for the NS5/3'UTR. In comparison, the two clusters of WN viruses in lineage I and the WN isolates in lineage II shared an average sequence identity of only 78% and 71% for the E gene and NS5/3'UTR, respectively. These results demonstrate that the sequences of some WN isolates are more closely related to the Australian KUN viruses than to other WN isolates.

The high degree of nucleotide sequence homology within clusters is consistent with the observed similarity of the amino acid sequences. The most notable variation in amino acid sequence in this study appears around the potential glycosylation site at amino acid 154 of the E protein (Figure 3). The Australian KUN viruses generally contain either the glycosylation motif NYS at this position or the sequence NYF, which abolishes glycosylation of the E protein. In contrast, the KUN virus SH183 has a 154N→K substitution, which also ablates the potential for glycosylation at this site. In comparison with the KUN prototype, the amino acids 159 (T→I, T→V, or T→Q) and 162 (A→T) of all the WN isolates in this study contain an amino acid substitution. The KUN isolate P1553 also differs from the KUN prototype at amino acid 159 (T→I). Two aberrant isolates, 63134Ent280 and WNFCG, incur a deletion of four amino acids (154 through 157), which also abolishes the glycosylation site.

Our results concur with those of Berthet et al. (16), who suggested the presence of signature motifs within the E gene that support the segregation of WN viruses into two lineages. These signature residues include the amino acid substitutions from lineage I→II as follows: 172A→S, 205T→S, and 210T→S. The amino acid substitution 208T→A holds true in general; however, two of the Indian isolates (lineage I) have K at this position and WNFCG (lineage II) has E. Of particular note is the substitution at amino acid 199. The Australian KUN isolates (199S) share the same amino acid as the lineage II WN viruses, while the lineage I viruses contain an N residue at this position. We have also identified an additional three signature motifs (I→II) at amino acids 128R→W, 129T→I, and 131L→Q. When we attempted to place the Malaysian KUN isolate within either lineage by using these signature motifs, the residues at 128, 129, 131, 172, and 208 were similar to those of lineage I viruses, but the residues at

Figure 3. Amino acid alignment of the region surrounding the potential glycosylation site of the E protein (shown in bold). KUN viruses not shown display the identical amino acid sequence as the prototype or the isolate OR205, depending on the glycosylation status of the virus. Alignment was performed with the Clustal W program.

205 and 210 were consistent with those of lineage II viruses. Residue 199 (D) was unlike any of the other viruses. The KOU isolate displayed more similarities with the lineage II WN viruses (residues 131, 172, 199, and 210) when signature motifs were compared. Residues 129 and 208 differed from viruses of both lineages.

We have identified signature motifs within the NS5 protein that correlate with the separation of the two lineages. Substitutions between lineages I→II include 860A→T, 869Q→H, 878I→V (except for the isolate MgAn798, which has 878I→L), and 899L→V (except for the isolate ArNa1047, which has 899L→I) (Figure 4). At amino acid 877, the lineage I WN viruses are separated again from the lineage II WN viruses with an A→S substitution; however, the KUN isolates (including MP502-66 from Malaysia) have the same motif as the lineage II WN viruses (877S). The amino acid substitution at 903 separates the Indian WN viruses (903S) from the WN and KUN viruses of both lineages (903T), instead grouping them with the Malaysian isolate and the KOU virus. Once again, the signature motifs cannot be used to classify the Malaysian isolate and KOU virus into either lineage.

Nucleotide sequences in the 3'UTR of the viruses included in this study had a highly variable region in both length and nucleotide sequence immediately downstream of the open reading frame stop codon (Figure 5). Deletions as well as point mutations were observed in this region, which varied from 38 (MgAn798) to 129 (ArNa1047) nt in length. The Australian KUN viruses displayed only point mutations when compared with the KUN prototype, except for the isolate P1553, which contained a 7-nt insertion, consistent with the WN viruses of lineage I. The long deletion in the nucleotide sequence immediately downstream of the stop codon of the WN prototype virus, WNFCG (53 nt), has been described (31); it is also present in the sequences of another two lineage II WN viruses analyzed in this study, Sarafend (53 nt) and MgAn798 (65 nt). The rest of the 3'UTR for these viruses was found to be highly conserved.

```
             860      869     877                     899 903
              ↓        ↓       ↓                       ↓   ↓
MRM61C       RARATWAENIQVAINQVRSIIGDEKYVDYMSSLKRYEDTTLVEDTVL
ArTB3573     ..................A............................
HB6343       ..................A............................
ArA1Dj       ..................A............................
NY99         ..................A............................
G2266        ..................A...E..........R...EV.....S..
G22886       ..................A...E..........R...EV.....S..
804994       ..................A...E..........R...EV.....S..
63134Ent280  .T........H........V..E..........R......V......
WNFCG        .T........H........V..E..........R......V......
Sarafend     .T........H........V..E.......G..R......V......
ArNa1047     .T........H........V..E.R.....G..R.....II......
MgAn798      .T........H........L..EG.........R......V......
MP502-66     ......................E..F.......R....I.....S..
KOUDakAad    .P........HA......A.......I....T.R....ASV...S..
JaOArS982    .S........YA......AV..K.N.....T..R....VLIQ..R.I
```

Figure 4. Amino acid alignment of the distal region of the NS5 protein. The KUN viruses not shown display a similar amino acid sequence to the prototype, except for a few minor point mutations not found within the signature motifs. Alignment was performed with the Clustal W program.

### Antigenic Analysis

The MAb 10A1, produced to the KUN isolate OR393 (26), reacted specifically with the Australian KUN isolates in ELISA and did not react with the KUN isolate from Malaysia (MP502-66) nor with KOU virus or any of the lineage I or lineage II WN viruses (Table 4). The MAb 546 (29), produced to the WN strain Eg101, reacted with all the lineage I and lineage II WN isolates except WN-Sarafend; it did not react with the KOU, KUN, or Malaysian viruses. The MAbs 2B2, produced to the KUN isolate MRM 16 (27), and 2B4, produced to the WN isolate H442 (28), reacted with all the isolates in the study, while the MAbs 3.67G and 3.91D, again produced to the KUN isolate OR393 (26), reacted with all the isolates except WN-Sarafend. The MAb 3.1112G, produced to the NS1 protein of KUN isolate OR393 (26), reacted with all isolates except KOU. The Mab binding patterns (Table 4) clearly digress and fail to differentiate KUN and WN isolates into two distinct groups. Instead, they define five distinct antigenic groups: Australian KUN viruses, Malaysian KUN virus, lineage I and lineage II WN viruses, WN-Sarafend, and KOU virus.

### Conclusion

The results of the phylogenic analysis in this report clearly illustrate that the KUN, WN, and KOU viruses make up a closely related group of viruses, which can be further subdivided into several subgroups on the basis of genetic and antigenic data. Previous phylogenic studies have also shown that KUN and WN viruses share a close relationship (16-18,21). This report however, further defines this relationship by using a comprehensive panel of both viruses. Also included in this study were several anomalous isolates, including an isolate from Southeast Asia (MP502-66), a laboratory-adapted WN strain of uncertain passage history and origin (Sarafend), and a flavivirus from West Africa (KOU), which has been shown to be closely related to the KUN/WN group of viruses.

The region sequenced in the E gene spans a glycosylation site that, although highly conserved among viruses of the JE antigenic subgroup, is absent from many KUN and WN isolates (16,26; Scherret JH, Khromykh AA, Mackenzie JS, Hall RA, unpub. data). While glycosylation at this site has been associated with neuroinvasiveness of WN isolates in mice (32,33), the biological significance of E protein glycosylation is still unclear. Indeed, sequence analysis of the E gene of WN viruses responsible for fatal outbreaks of encephalitis in Romania (Rom 96) and New York (NY99) showed that only the latter contained a potential glycosylation site, casting doubt on the importance of E protein glycosylation in viral pathogenesis. However, our studies and those of others have shown that limited passage of WN and KUN viruses in some cell types can alter the glycosylation status of the E protein and that analysis of passaged viral isolates should be interpreted with caution

Figure 5. Nucleotide sequence alignment of the 3'UTR (untranslated region) proximal to the open reading frame stop codon (shown in bold) showing distinctive insertions or deletions. Alignment was performed with the Clustal W program.

(33; Scherret JH, Khromykh AA, Mackenzie JS, Hall RA, unpub. data).

The 3'UTR of flaviviruses ranges in length from 400 nt to 600 nt and is thought to play a crucial role in the initiation and regulation of viral translation, replication, and assembly. It includes a potential stable secondary RNA structure at its terminus (2,34-38), and upstream it contains several domains that appear to be conserved among mosquito-borne flaviviruses (2,39, 40). Men et al. (41) have suggested that deletions in the distal 80 nt to 90 nt would most likely lead to disruption of the stem-loop and loss of viability. In contrast, the region sequenced in this study contains highly variable regions suitable for genetic classification and analysis of the relationships among viruses, which had been subjected to deletions or insertions or both during evolution (17).

Phylogenetic trees constructed from sequence data from both regions identified two major lineages, consistent with previous reports (16,18,21). These two lineages did not separate the KUN isolates from the WN isolates; rather, they emphasized the close link between KUN and WN viruses of lineage I. Nevertheless, within lineage I, the Australian KUN isolates formed a tight cluster with an average nucleotide divergence of 6% for the E gene and 10% for the NS5/3'UTR. In contrast, the WN isolates were spread between the two lineages in three clusters, with a divergence of up to 30.6% for sequences of the E gene and 28.3% for sequences of the NS5/3'UTR. Signature motifs in the deduced amino acid sequences of the E and NS5 proteins also support the separation of the viruses into two lineages.

The virus from Malaysia, KUN MP502-66, and the African virus, KOU, pose a conundrum as to their relationship with the WN and KUN group of viruses. Statistical support for clustering with either of the WN lineages was poor, suggesting that they represent two single-isolate lineages. Although our previous findings suggested that the Malaysian KUN isolate may represent an evolutionary link between the KUN and WN viruses (17), the lack of sequence identity between KUN MP502-66 and the KUN/WN group of viruses in our study suggests that these viruses have evolved separately from a common ancestor.

Table 4. Binding patterns of anti-KUN and anti-WN monoclonal antibodies to virus isolates in enzyme-linked immunosorbent assay (ELISA)[a]

| | Monoclonal antibodies (MAb) | | | | | | |
|---|---|---|---|---|---|---|---|
| Virus | 10A1 | 546 | 2B2 | 2B4 | 3.91D | 3.67G | 3.1112G |
| KUN[b] | +[c] | - | + | + | + | + | + |
| KUN MP502-66 | - | - | + | + | + | + | + |
| WN[d] | - | + | + | + | + | + | + |
| WN Sarafend | - | - | + | + | - | - | + |
| KOU | - | - | + | + | + | + | - |

[a]Infected C6/36 cell monolayers in 96-well plates were fixed with acetone and used as the antigen in the ELISA.
[b]All Australian KUN isolates exhibited identical MAb binding patterns.
[c]A result was considered positive if consecutive twofold dilutions of MAb produced an OD >0.25 and at least twice that shown on uninfected cells.
[d]All West Nile isolates except Sarafend produced identical MAb binding patterns.

The binding patterns of MAbs to KUN and WN isolates did not differentiate these viruses into the same phylogenetic lineages observed in the dendrograms, although they did support the sequencing results by identifying the Australian KUN viruses, the Malaysian KUN virus, and KOU virus as distinct antigenic groups. The WN-specific MAb used in this study, 546, could not distinguish subgroups within the WN group of viruses; however, Besselaar and Blackburn (28) and Damle et al. (42) have differentiated Indian WN isolates from lineage I South African strains by using MAbs, consistent with the earlier studies of Hammam et al. (43,44). These findings support our sequence data, which show tight clustering of the Indian isolates on a separate branch from other WN isolates in the phylogenetic trees (Figures 1 and 2). Additional MAbs to the E protein of WN viruses may be required to differentiate between lineage I and lineage II viruses.

The unique binding pattern of anti-E MAbs to the Sarafend WN isolate is difficult to explain in light of the E gene sequencing results and amino acid alignments, which show that this virus is similar to other lineage II viruses. However, Sarafend also differs from other WN viruses in the way that it buds from the cell membrane of infected cells (45). Sequencing of the entire prM and E genes of this virus may identify the basis for structural differences in the envelope heterodimer that account for the loss of MAb binding sites and unusual virion maturation.

Phylogenetic analyses enable more precise determination of the relationships among similar viruses and consequently aid in identifying the origin of unknown viruses in subsequent outbreaks. The importance of defining the relationship between the KUN and WN viruses was emphasized during the 1999 outbreak of viral encephalitis in New York City (46,47). Until recently, WN and KUN had been classified as distinct virus types in the *Flavivirus* genus. However, the latest report by the International Committee on Taxonomy of Viruses (25) recognized that KUN and WN should not be classified as two separate species and designated KUN as a subtype of WN. Our results suggest that this definition requires further consideration. The species should perhaps be further subdivided into at least six subtypes on the basis of the clusters of viruses displayed in the phylogenetic trees. Subtypes would then include lineage II WN group, Indian WN group, Australian KUN group, lineage I WN group, Malaysian group, and KOU group.

Indeed, the assessment of viruses from each subgroup for transmissibility by the major mosquito vectors of each geographic region and relative virulence and amplification in primate, equine, and avian species will provide valuable information on the likelihood and possible consequences of the spread of these viruses to new geographic regions. Additional studies of cross-protection between subgroups by natural infection or immunization with vaccines derived from these viruses and the specificity and sensitivity of serologic and molecular assays for each subgroup in monitoring and diagnostic applications will be useful in defining control strategies.

### Acknowledgments

We thank Robert Lanciotti for valuable scientific discussions regarding the New York outbreak, Terry Besselaar for supplying monoclonal antibodies, and J.P. Thakare and S.S. Gogate for providing the isolates from India. We also appreciate comments on the manuscript by Helle Bielefeldt-Ohmann and technical assistance from Petra Sedlak.

This work was supported by a research grant from the National Health and Medical Research Council of Australia.

Dr. Scherret is a postdoctoral fellow with the World Health Organization Collaborating Center for Tropical Diseases, University of Texas Medical Branch, Galveston, Texas. Her dissertation concerned the molecular epidemiology and biology of Kunjin and West Nile viruses. Her research interests focus on viral hemorrhagic diseases, including dengue and Oropouche fever.

### References

1. Heinz FX, Collett MS, Purcell RH, Gould EA, Howard CR, Houghton M, et al. Family *Flaviviridae*. In: van Regenmortel MHV, Fauquet CM, Bishop DHL, Carstens EB, Estes MK, Lemon SM, et al., editors. Virus taxonomy: Classification and nomenclature of viruses. 7th Report of the International Committee for the Taxonomy of Viruses. San Diego: Academic Press, 2000; p. 859-78.
2. Chambers TJ, Hahn CS, Galler R, Rice CM. Flavivirus genome organization, expression, and replication. Annu Rev Microbiol 1990;44:649-88.
3. Kuno G, Chang GJJ, Tsuchiya R, Karabatsos N, Cropp CB. Phylogeny of the genus Flavivirus. J Virol 1998;72:73-83.
4. Hayes C. West Nile Fever. In: Monath TP, editor. The arboviruses: epidemiology and ecology. Vol. III. Boca Raton (FL): CRC Press; 1988. p. 59-88.
5. Marshall ID. Murray Valley and Kunjin encephalitis. In: Monath TP, editor. The arboviruses: epidemiology and ecology. Vol. III. Boca Raton (FL): CRC Press, 1988; p. 151-89.
6. Monath TP, Heinz FX. Flaviviruses. In: Fields BN, Knipe DM, Howley PM, editors. Fields virology. 3rd ed. Philadelphia: Lippincott-Raven, 1996; p. 961-1034.
7. Westaway EG. The neutralization of arboviruses. II. Neutralization in heterologous virus-serum mixtures with four group B arboviruses. Virology 1965;26:528-37.
8. De Madrid AT, Porterfield JS. The flaviviruses (group B arboviruses): a cross-neutralization study. J Gen Virol 1974;23:91-6.
9. Calisher CH, Karabatsos N, Dalrymple JM, Shope RE, Porterfield JS, Westaway EG, et al. Antigenic relationships between flaviviruses as determined by cross-neutralization tests with polyclonal antisera. J Gen Virol 1989;70:37-43.
10. Coia G, Parker MD, Speight G, Byrne ME, Westaway EG. Nucleotide and complete amino acid sequences of Kunjin virus: Definitive gene order and characteristics of the virus-specified proteins. J Gen Virol 1988;69:1-21.
11. Castle E, Nowak T, Leidner U, Wengler G, Wengler G. Sequence analysis of the viral core protein and membrane associated proteins V1 and NV2 of the flavivirus West Nile virus and of the genome sequence for these proteins. Virology 1985;145:227-36.
12. Wengler G, Castle E, Leidner U, Nowak T. Sequence analysis of the membrane protein V3 of the flavivirus West Nile virus and of its gene. Virology 1985;147:264-74.
13. Castle E, Leidner U, Nowak T, Wengler G. Primary structure of the West Nile flavivirus genome region coding for all nonstructural proteins. Virology 1986;149:10-26.
14. Lobigs M, Weir RC, Dalgarno L. Genetic analysis of Kunjin virus isolates using HAE III and TAQ I restriction digests of single-stranded cDNA to virion RNA. Aust J Exp Biol Med Sci 1986;64:185-96.
15. Flynn LM, Coelen RJ, Mackenzie JS. Kunjin virus isolates of Australia are genetically homogeneous. J Gen Virol 1989;70:2819-24.
16. Berthet FX, Zeller HG, Drouet MT, Rauzier J, Digoutte JP, Deubel V. Extensive nucleotide changes and deletions within the envelope glycoprotein gene of Euro-African West Nile viruses. J Gen Virol 1997;78:2293-7.

17. Poidinger M, Hall RA, Mackenzie JS. Molecular characterisation of the Japanese encephalitis serocomplex of the Flavivirus genus. Virology 1996;218:417-21.
18. Lanciotti RS, Roehrig JT, Deubel V, Smith J, Parker M, Steele K, et al. Origin of the West Nile virus responsible for an outbreak of encephalitis in the northeastern U.S. Science 1999;286:2333-7.
19. Tsai TF, Popovici F, Cernescu C, Campbell GL, Nedelcu NI. West Nile encephalitis epidemic in southeastern Romania. Lancet 1998;352:767-71.
20. Savage HM, Ceianu C, Nicolescu G, Karabatsos N, Lanciotti R, Vladimirescu A, et al. Entomologic and avian investigations of an epidemic of West Nile fever in Romania in 1996, with serological and molecular characterization of a virus isolate from mosquitoes. Am J Trop Med Hyg 1999;61:600-11.
21. Jia XY, Briese T, Jordan I, Rambaut A, Chi HC, Mackenzie JS, et al. Genetic analysis of the West Nile New York 1999 encephalitis virus. Lancet 1999;354:1971-2.
22. Sellner LN, Coelen RJ, Mackenzie JS. A one-tube, one manipulation RT-PCR reaction for detection of Ross River virus. J Virol Methods 1992;40:255-64.
23. Pierre V, Drouet MT, Deubel V. Identification of mosquito-borne flavivirus sequences using universal primers and reverse transcription/polymerase chain reaction. Res Virol 1994;145:93-104.
24. Thompson JD, Higgins DG, Gibson TJ. CLUSTAL W: Improving the sensitivity of progressive multiple sequence alignment through sequence weighting, position-specific gap penalties and weight matrix choice. Nucleic Acids Res 1994;22:4673-80.
25. Felsenstein J. PHYLIP—Phylogeny Inference Package (Version 3.2). Cladistics 1989;5:164-6.
26. Adams SC, Broom AK, Sammels LM, Hartnett AC, Howard MJ, Coelen RJ, et al. Glycosylation and antigenic variation among Kunjin virus isolates. Virology 1995;206:49-56.
27. Hall RA, Burgess GW, Kay BH, Clancy P. Monoclonal antibodies to Kunjin and Kokobera viruses. Immunol Cell Biol 1990;69:47-9.
28. Besselaar TG, Blackburn NK. Antigenic analysis of West Nile virus strains using monoclonal antibodies. Arch Virol 1988;99:75-88.
29. Gould EA, Buckley A, Higgs S, Gaidamovich S. Antigenicity of flaviviruses. Arch Virol Suppl 1 1990;137-52.
30. Sumiyoshi H, Mori C, Morita K, Kuhara S, Kondou J, Kukushi Y, et al. Complete nucleotide sequence of the Japanese encephalitis virus genome RNA. Virology 1987;161:497-510.
31. Khromykh AA, Westaway EG. Completion of Kunjin virus RNA sequence and recovery of an infectious RNA transcribed from stably cloned full-length cDNA. J Virol 1994;68:4580-8.
32. Halevy M, Akov Y, Ben-Nathan D, Kobiler D, Lachmi B, Lustig S. Loss of active neuroinvasiveness in attenuated strains of West Nile virus: pathogenicity in immunocompetent and SCID mice. Arch Virol 1994;137:355-70.
33. Chambers TJ, Halevy M, Nestorowicz A, Rice CM, Lustig S. West Nile virus envelope proteins: nucleotide sequence analysis of strains differing in mouse neuroinvasiveness. J Gen Virol 1998;79:2375-80.
34. Brinton MA, Fernandez AV, Dispopto JH. The 3'-nucleotides of flavivirus genomic RNA form a conserved secondary structure. Virology 1986;153:113-21.
35. Proutski V, Gaunt MW, Gould EA, Holmes EC. Secondary structure of the 3'-untranslated region of Yellow Fever virus: Implications for virulence, attenuation and vaccine development. J Gen Virol 1997;78:1543-9.
36. Rice CM, Lenches EM, Eddy SR, Shin SJ, Sheets RL, Strauss JH. Nucleotide sequence of yellow fever virus: Implications for Flavivirus gene expression and evolution. Science 1985; 229:726-33.
37. Takegam T, Washizu M, Yasui K. Nucleotide sequence at the 3' end of Japanese encephalitis virus genomic RNA. Virology 1986;152:483-6.
38. Wengler G, Castle E. Analysis of structural properties which possibly are characteristic for the 3' terminal sequence of the genome RNA of flaviviruses. J Gen Virol 1986;67:1183-8.
39. Hahn CS, Hahn YS, Rice CM, Lee E, Dalgarno L, Strauss EG, et al. Conserved elements in the 3' untranslated region of the flavivirus RNAs and potential cyclization sequences. J Mol Biol 1987;198:33-41.
40. Deubel V, Kinney RM, Trent DW. Nucleotide sequence and deduced amino-acid sequence of the non-structural proteins of dengue type 2 virus, Jamaica genotype: comparative analysis of the full-length genome. Virology 1988;165:234-44.
41. Men R, Bray M, Clark D, Chanock RM, Lai CJ. Dengue Type 4 virus mutants containing deletions in the 3' noncoding region of the RNA genome: Analysis of growth restriction in cell culture and altered viremia pattern and immunogenicity in Rhesus monkeys. J Virol 1996;70:3930-7.
42. Damle RG, Yeolekar LR, Rao BL. Strain analysis and epitope mapping of West Nile virus using monoclonal antibodies. Acta Virol 1998;42:389-95.
43. Hammam HM, Clark DH, Price WH. Antigenic variation of West Nile virus in relation to geography. Am J Epidemiol 1965;82:40-55.
44. Hammam HM, Price WH. Further observations on geographic variation in the antigenic character of West Nile and Japanese B viruses. Am J Epidemiol 1966;83:113-22.
45. Ng ML, Howe J, Sreenivasan V, Mulders JJ. Flavivirus West Nile (Sarafend) egress at the plasma membrane. Arch Virol 1994;137:303-13.
46. Briese T, Jia XY, Huang C, Grady LJ, Lipkin WI. Identification of a Kunjin/West Nile-like flavivirus in brains of patients with New York encephalitis. Lancet 1999; 354:1261-2.
47. Fine A, Layton M, Miller J, Cimini D, Vargas MC, Inglesby A, et al. Update: West Nile virus encephalitis, New York, 1999. MMWR Morb Mortal Wkly Rep 1999;48:944-6.
